# Flotillin-2 is an acrosome-related protein involved in mouse spermiogenesis

**DOI:** 10.7555/JBR.26.20120030

**Published:** 2012-07-02

**Authors:** Yibo Wu, Xin Chen, Shuai Wang, Min Jiang, Bo Zheng, Quan Zhou, Ye Bi, Zuomin Zhou, Xiaoyan Huang, Jiahao Sha

**Affiliations:** State Key Laboratory of Reproductive Medicine, Department of Histology and Embryology, Nanjing Medical University, Nanjing, Jiangsu 210029, China.

**Keywords:** flotillin-2, Golgi apparatus, RNA interference, acrosome biogenesis

## Abstract

Spermatogenesis is a complex process of terminal differentiation by which mature sperms are generated, and it can be divided into three phases: mitosis, meiosis and spermiogenesis. In a previous study, we established a series of proteomic profiles for spermatogenesis to understand the regulation of male fertility and infertility. Here, we further investigated the localization and the role of flotillin-2 in spermiogenesis. Flotillin-2 expression was investigated in the testis of male CD1 mice at various developmental stages of spermatogenesis by using Western blotting, immunohistochemistry and immunofluorescence. *Flotillin-2* was knocked down *in vivo* in three-week-old male mice using intratesticular injection of small inhibitory RNA (siRNA), and sperm abnormalities were assessed three weeks later. Flotillin-2 was expressed at high levels in male germ cells during spermatogenesis. Flotillin-2 immunoreactivity was observed in pachytene spermatocytes as a strong dot-shaped signal and in round spermatids as a sickle-shaped distribution ahead of the acrosome. Immunofluorescence confirmed flotillin-2 was localized in front of the acrosome in round spermatids, indicating that flotillin-2 was localized to the Golgi apparatus. Knockdown of *flotillin-2* *in vivo* led to a significant increase in head sperm abnormalities isolated from the cauda epididymis, compared with control siRNA-injected testes. This study indicates that flotillin-2 is a novel Golgi-related protein involved in sperm acrosome biogenesis.

## INTRODUCTION

Spermiogenesis, which occurs in the upper layers of the seminiferous epithelium, is the final stage of spermatogenesis, during which mature and motile spermatozoa are formed by condensation of nuclear chromatin, elongation of the nucleus, formation of the acrosome, formation of a single flagellum and loss of residual cytoplasm[1],[2]. Biogenesis of the acrosome is a highly orchestrated process involving the delivery of protein and membrane from the Golgi apparatus to the developing acrosome. The Golgi complex initially contains round small vesicles, which fuse to form a large acrosomal vesicle. Along with a sperm nucleus of variable length, acrosomal vesicles spread radially over one-third and, eventually, one half of the compacting nucleus. After acrosomal protein production is completed, the Golgi apparatus separates from the acrosomal vesicle and begins to migrate to the caudal portion of elongating spermatids. Finally, the Golgi saccules appear to be destined for use in the cytoplasmic droplet[3]^,^[4].

The Golgi apparatus is a very important organelle in acrosome formation. At present, four Golgi proteins have been reported in pachytene spermatocytes and round elongating spermatids, including giantin, β-coat protein (β-COP), Golgin-97 and Golgin-95/GM130. These proteins are also detected in the membranes surrounding the acrosome until the late cap-step spermatid stage, and finally are shed in the cytoplasmic droplet[3],[5],[6]. In addition, many other Golgi−related proteins have been identified to be continuously expressed during acrosome biogenesis, including Golgi−associated PDZ− and coiled−coil motif−containing protein (GOPC), Golgin A3 (GOLGA3), translinassociated factor X (TRAX) and spermatogenesis associated 16 (SPATA16)[7]−[10]. However, the mechanism where Golgi−derived proacrosomal vesicles are mobilized toward a precise site during acrosome biogenesis is not wholly understood[11].

In our previous studies, we used different proteomic technologies to obtain a global view of protein expression in whole mouse and human testes[12],[13]. Subsequently, the whole-protein expression profiles of different germ cells, including tetraploid and haploid germ cells, were characterized[14],[15]. These investigations have established a series of proteomic profiles associated with spermatogenesis and produced several important protein lists, which have helped to understand the regulation of male fertility and infertility[16]. Based on literature and the specificity of available antibodies, we chose several proteins from these lists for further investigation. Flotillin-2 is one such protein, and a high quality antibody is available which recognizes a single 48 kDa band in the mouse testis. In this study, we explored whether flotillin-2 was a novel Golgi-related protein involved in acrosome biogenesis and normal spermiogenesis. We identified that flotillin-2 was located ahead of the acrosome during spermiogenesis, and that knockdown of *flotillin-2* expression led to abnormal sperm head morphology.

## MATERIALS AND METHODS

### Animals and cell culture

Male CD-1 mice (0 d/4 d/1 w/10 d/2 w/3 w/4 w/5 w old and adult) were obtained from the Animal Center of Nanjing Medical University (Nanjing, Jiangsu, China). All experiments were performed in accordance with the protocols approved by the Institutional Animal Care and Use Committee and followed the Guide for the Care and Use of Laboratory Animal of Nanjing Medical University.

The immortalized mouse spermatocyte cell line (GC2-spd; CRL-2196) was purchased from American Type Culture Collection (ATCC; Manassas, VA, USA) and cultured in Dulbecco's modified Eagle's medium (DMEM; Gibco BRL, Grand Island, NY, USA) at 37°C in a 5% CO_2_ atmosphere.

### Sample preparation and protein extraction

The testes from male CD-1 mice were solubilized in lysis buffer [7 mol/L urea, 2 mol/L thiourea, 4% (*W/V*) CHAPS, and 2% (*W/V*) DTT] in the presence of 1% (*W/V*) protease inhibitor cocktail (Pierce Biotechnology, Rockford, IL, USA), and then homogenized and sonicated. The mixture was placed on a shaker at 4°C for 1 h, and insoluble matter was removed by centrifugation at 40,000 *g* for 1 h at 4°C. GC2-spd cells were solubilized in lysis buffer. The protein concentration of each sample was determined by the Bradford Protein Assay[17] using bovine serum albumin (BSA) as a standard.

### Western blot analysis

Samples containing 100 μg protein were electrophoresed on 12% SDS polyacrylamide gels, transferred to PVDF membranes (GE Healthcare, San Francisco, CA, USA), blocked in TBS containing 5% non-fat milk powder for 1 h and incubated overnight with anti-flotillin-2 (Sigma, St. Louis, MO, USA) and anti-β-tubulin (Abcam, Cambridge, MA, USA) antibodies in TBS containing 5% non-fat milk powder. The expression of β-tubulin was used as a loading control. The membranes were washed, incubated for 1 h with horseradish peroxidase (HRP)-conjugated goat anti-rabbit IgG (Beijing Zhongshan Biotechnology Co., Beijing, China) and the protein bands were detected using the enhanced chemiluminescence (ECL) Western Blotting detection kit and AlphaImagerTM (GE Healthcare, San Francisco, CA, USA).

### Immunohistochemistry

Bouin's solution-fixed paraffin-embedded sections from mouse testis were immunostained as previously described[17]. In brief, after quenching endogenous peroxidase activity, the sections were blocked using blocking serum and incubated overnight at 4°C with anti-flotillin-2 antibody, and then incubated with HRP-conjugated secondary antibody (Beijing ZhongShan Biotechnology). Immunoreactivity was visualized (brown) using diaminobenzidine and the sections were mounted for bright field microscopy (ZEISS Fluorescent Microsystems, Göttingen, Germany). To confirm the specificity of flotillin-2 antibody, negative controls were processed in an identical manner; however, the primary antibody was replaced with normal IgG.

### Indirect immunofluorescence

Mouse testis sections were deparaffinized twice in xylene for 15 min, rehydrated using a descending gradient ethanol series and washed twice with triple-distilled water for 5 min. Antigen retrieval was performed using 2% EDTA solution, and the sections were blocked in blocking serum for 2 h at room temperature, incubated overnight with primary flotillin-2 antibody (1:300) at 4°C and then incubated with FITC-labeled secondary antibody (1:200; Beijing Zhongshan Biotechnology) for 1 h at room temperature. Negative controls were prepared by replacing the primary antibody with normal rabbit IgG (Santa Cruz Biotechnology, Santa Cruz, CA, USA). Finally, the acrosome was stained using 1 μg/mL peanut agglutinin (PNA; L3766; Sigma, St. Louis, MO, USA) for 2 h at room temperature. The slides were washed three times for 5 min with PBS and the nuclei were stained with 5 μg/mL Hoechst (H33342; Sigma) for 30 s.

### Intratesticular injection of siRNA against flotillin-2

Double-stranded Stealth siRNAs against flotillin-2 (Invitrogen; Catalog No. MSS204353, MSS204354 and MSS204355) were diluted to a final concentration of 20 μM and stored at –20°C. According to Invitrogen's instructions, the efficiency of flotillin-2 knockdown by the three siRNAs was verified in GC-2-spd cells at 72 h using Western blotting, and the highest efficiency siRNA (MSS204354) was used for all *in vivo* studies.

Intratesticular injections were performed as previously described[15],[18]. Briefly, 3-week old male mice were anesthetized with sodium pentobarbital, and then the testes were exteriorized through an approximately 5 mm midline abdominal incision. Approximately 3–5 µL of siRNA mixed with indicator (0.4% trypan blue) was injected into the seminiferous tubules via rete testis injection. Control testes were injected with a negative control siRNA (Invitrogen; Catalog No. 12935-400). After injection, the testes were replaced in the abdomen, the incisions were sutured and the mice were allowed to recover and housed for another 3 weeks before euthanasia and classification of epididymal sperm morphology. To verify the efficiency of RNAi *in vivo*, flotillin-2 protein expression was quantified by Western blotting analysis of seminiferous tubule lysate 48 h after injection of siRNA.

### Epididymal sperm morphology classification

Three w after injection of siRNAs, spermatozoa were collected from the cauda epididymis, and then spread on slides, fixed in 4% paraformaldehyde, and stained with hematoxylin and eosin (H&E) and 1 μg/mL PNA for 2 h at room temperature before morphological observation. Deformities were classified as previously described[19]. Acrosome abnormalities were assessed using PNA staining. Differences between the groups were evaluated using Student's *t* test. *P* values less than 0.05 were considered significant.

## RESULTS

### The expression of flotillin-2 during mouse spermatogenesis

**Fig. 1:**
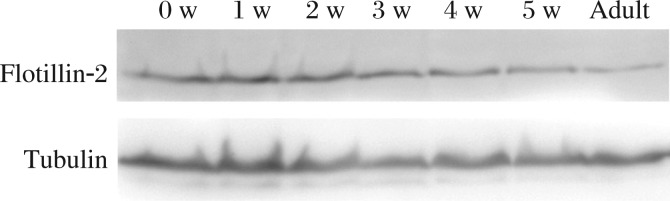
Western blotting analysis of flotillin-2 protein expression at different developmental stages in the mouse testis. Flotillin-2 is expressed in the testis during the first wave of spermatogenesis. Similar results were observed in triplicate experiments.

In a previous proteomic study, we identified a series of proteins involved in mouse spermatogenesis, including flotillin-2. Firstly, we verified the expression of flotillin-2 in adult mouse testis using Western blotting. Flotillin-2 was detected as a 48 kDa band in mouse adult testis (***Fig. 1***). In order to explore the expression of flotillin-2 during the first wave of spermatogenesis, we used proteins isolated from the testis of 0, 1, 2, 3, 4 and 5 weeks old mice. We observed that flotillin-2 was expressed throughout the first wave of spermatogenesis while the expression level was decreased along with the increase of age (***Fig. 1***).

### The localization of flotillin-2 during mouse spermatogenesis

**Fig. 2:**
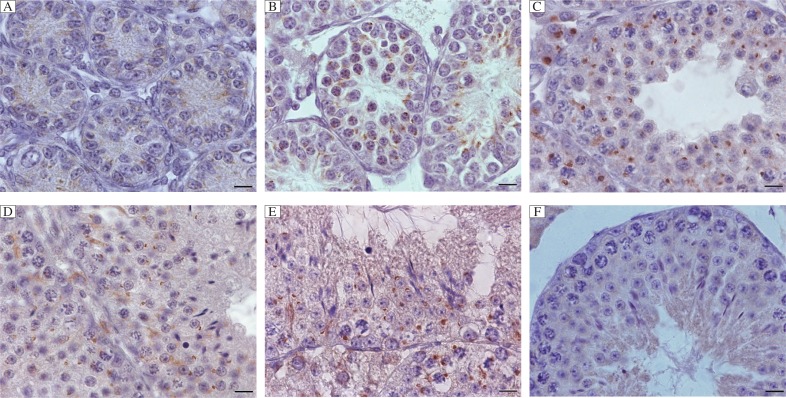
Immunohistochemical localization of flotillin-2 in the mouse testis at different developmental stages. Spematogonia in the testis of 4 d (A) and primary spermatocytes in the testis of 10 d (B) old mice displayed weak flotillin-2 immunoreactivity. A flotillin-2 dot-shaped immunoreactive signal was obvious in the pachytene spermatocytes of 3 weeks old mice (C), and as the round spermatids appeared, the signal elongated ahead of the acrosome. Flotillin-2 immunoreactivity was similar in the testis of 4 weeks old mice (D) and in adult mice (E), it was observed at high levels in pachytene spermatocytes as a strong dot-shaped signal and also in round spermatids as a sickle-shaped distribution in the acrosome. Control (F) showed negative signal. All scale bars are 10 µm.

To understand biological function of flotillin-2 during mouse spermatogenesis, we determined the localization of flotillin-2 in adult mouse testis using immunohistochemistry. Flotillin-2 was expressed in germ cells during spermiogenesis, and was observed at high levels in pachytene spermatocytes as a strong dot-shaped signal and also in round spermatids as a sickle-shaped distribution in the acrosome (***Fig. 2***).

At d 4, there were only spermatogonia and Sertoli cells in seminiferous tubules and then primary spermatocytes were present at d 10 of development. Three weeks signified the onset of spermiogenesis and by 4 weeks, elongating spermatids were observed[20],[21]. In order to investigate the precise localization of flotillin-2, we analyzed testis from 4 d, 10 d, 3 weeks and 4 weeks old mice using immunohistochemistry. Spematogonia in the testis of 4 d old mice and primary spermatocytes in the testis of 10 d old mice displayed weak flotillin-2 immunoreactivity. A flotillin-2 dot-shaped immunoreactive signal was obvious in the pachytene spermatocytes of 3 weeks old mice. As round spermatids appeared, the signal elongated ahead of the acrosome. Flotillin-2 immunoreactivity was similar in the testis of 4 weeks old mice and adult mice (***Fig. 2***).

### Flotillin-2 may be a Golgi-related protein during acrosome biogenesis

**Fig. 3:**
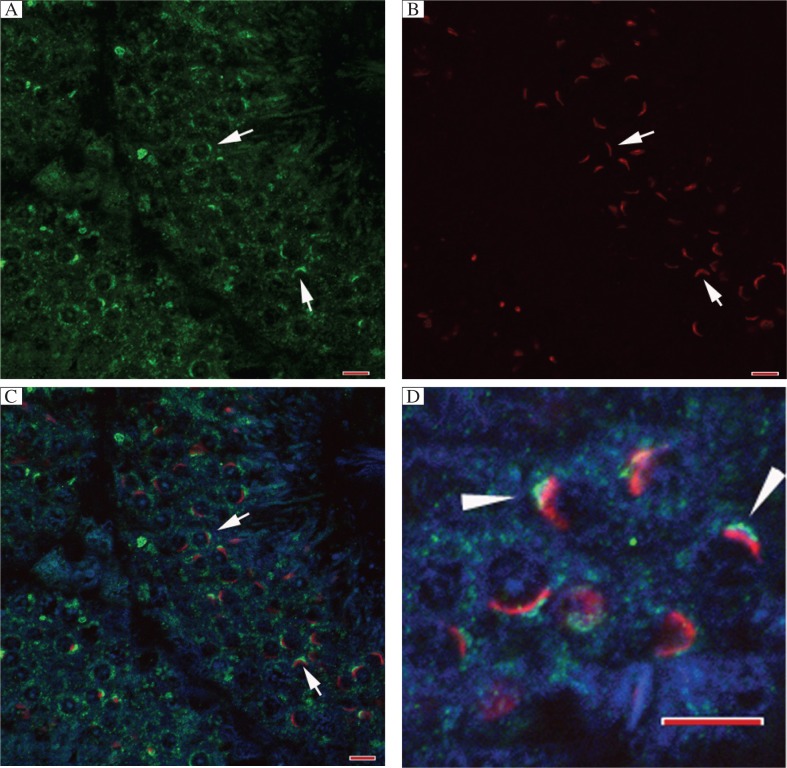
Flotillin-2 is located ahead of the developing acrosome. Immunofluorescent analysis of flotillin-2 in the testis of adult mouse. A: Flotillin-2 (green); B: PNA (red); C: merge; D: Higher magnification image. All scale bars are 10 μm.

Immunohistochemistry indicated that flotillin-2 may be associated with acrosome biogenesis. To clarify the localization of flotillin-2 with respect to the acrosome, we used PNA to label the outer acrosomal membrane[22] and preformed indirect immunofluorescence for flotillin-2. Flotillin-2 was located ahead of the outer acrosomal membrane in spermatids undergoing acrosome biogenesis (***Fig. 3A*** to***3D***). As the Golgi apparatus may be located ahead of the outer acrosomal membrane[3],[23], flotillin-2 may be a Golgirelated protein during acrosome biogenesis.

### Knockdown of flotillin-2 during spermiogenesis

**Fig. 4:**
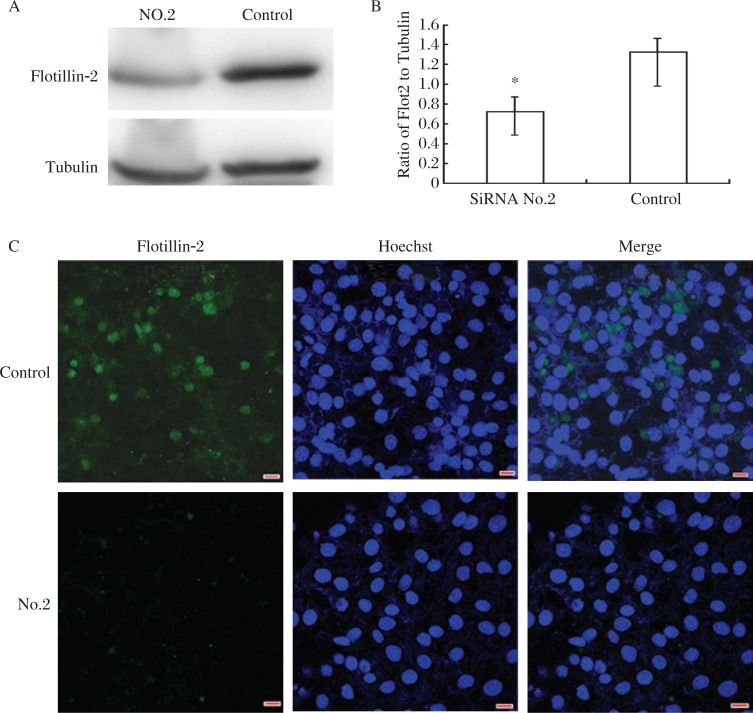
Efficiency of flotillin-2 siRNAs in GC2-spd cells. Western blotting analysis(A and B) of the effect of siRNA (MSS204354; Invitrogen) on flotillin-2 protein expression at 72 h. The data represent an average of three independent experiments, and are expressed as fold changes relative to the negative control. C: Immunofluorescent analysis of the effect of siRNA (MSS204354) on flotillin-2 protein expression at 72 h. All scale bars are 10 µm. Similar results were observed in three independent experiments.

Round spermatids begin to appear in the testis of 3 weeks old mice, which then undergo spermiogenesis[21]. To further investigate the function of flotillin-2, we performed RNAi in 3 weeks old male mice to observe whether knockdown of flotillin-2 would affect sperm morphology during the first wave of spermiogenesis. Western blotting and immunofluorescence demonstrated that MSS204354 siRNA (No. 2 siRNA) had the highest level of protein repression in GC2-spd cells at 72 h, and this siRNA was used for all subsequent analysis (***Fig. 4A, 4B*** and ***4C***).

**Fig. 5:**
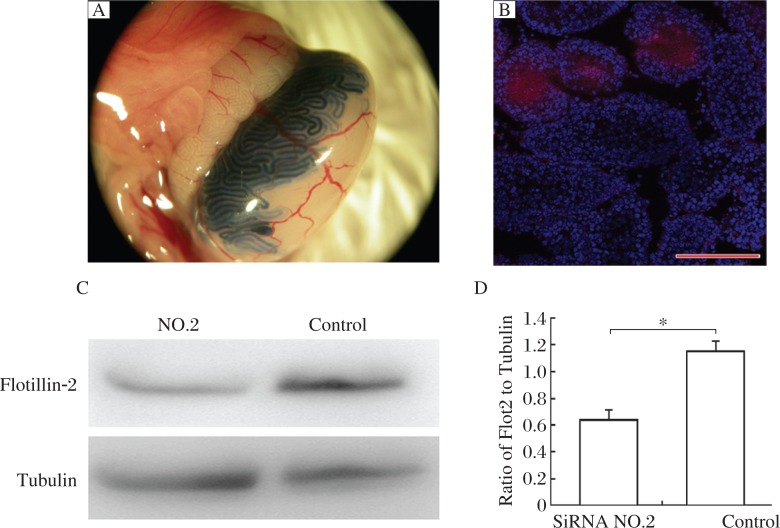
Efficiency of flotillin-2 siRNAs *in vivo*. A: Trypan blue staining was used as a marker of siRNA injection; 30–40% of the seminiferous tubules were injected with siRNA. B: Trypan blue staining (Red) showed siRNA injection did not lead to seminiferous tubule damage. Scale bar is 10 µm. C:Western blotting analysis of flotillin-2 protein expression in 3 week old mouse testis 48 h after injection of flotillin-2 siRNA. D: Quantification of Western blotting analysis. Data are the mean fold change relative to the control siRNA-injected mice from three independent experiments.

The seminiferous tubules of 3 weeks old male mice were injected with No. 2 siRNA against flotillin-2, which introduced siRNA into approximately 30-40% of the seminiferous tubules without causing seminiferous tubule damage (***Fig. 5A*** and ***5B***). To further verify the efficiency of RNAi *in vivo*, we collected the seminiferous tubules from some mice 48 h after injection of siRNA using trypan blue staining. Western blotting of seminiferous tubule lysate demonstrated that flotillin-2 protein was consistently suppressed 48 h after injection of siRNA (***Fig. 5C*** and ***5D***).

**Fig. 6:**
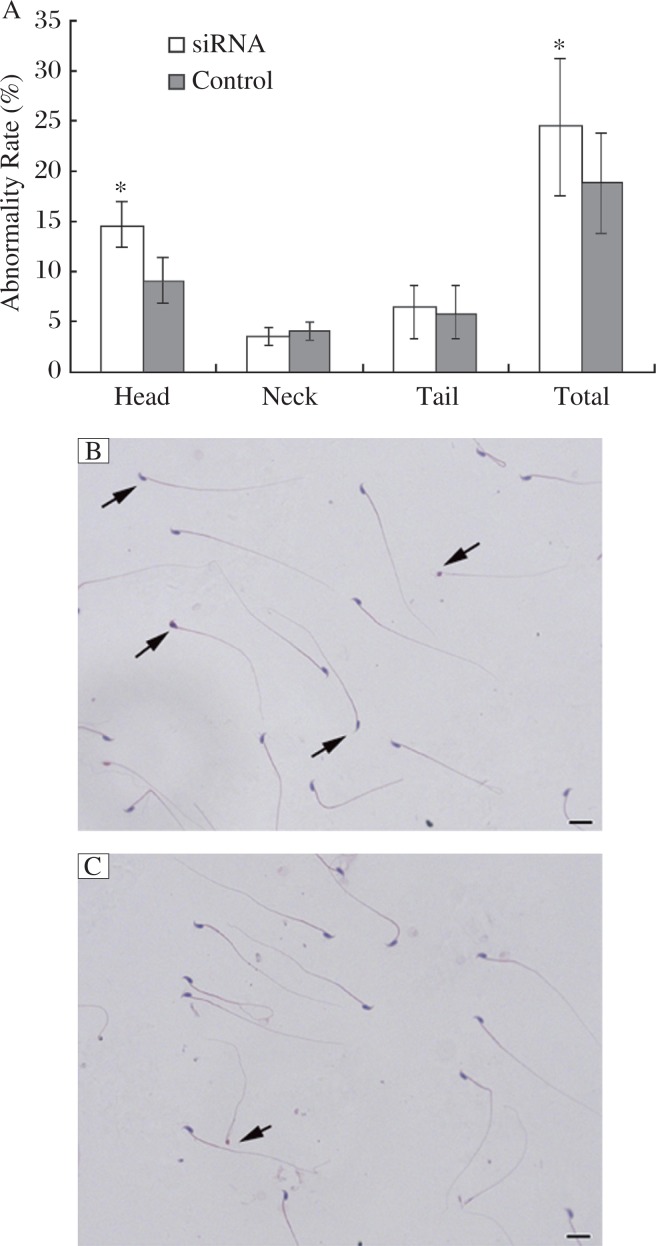
Knockdown of flotillin-2 *in vivo* affects sperm morphology in the cauda epididymis. The testes of three weeks old mice were injected with flotillin-2 siRNA and sperm were isolated from the cauda epididymis three weeks later. A: Sperm abnormalities. The ratios were subjected to arcsine square root transformation prior to Student's *t* test; *P < 0.05. Representative images of the shape of sperm from the cauda epididymis of mice injected with flotillin-2 siRNA (B) or control siRNA (C). The arrows indicate sperm with an abnormal shape. All scale bars are 10 µm. Similar results were observed in three independent experiments.

**Fig. 7:**
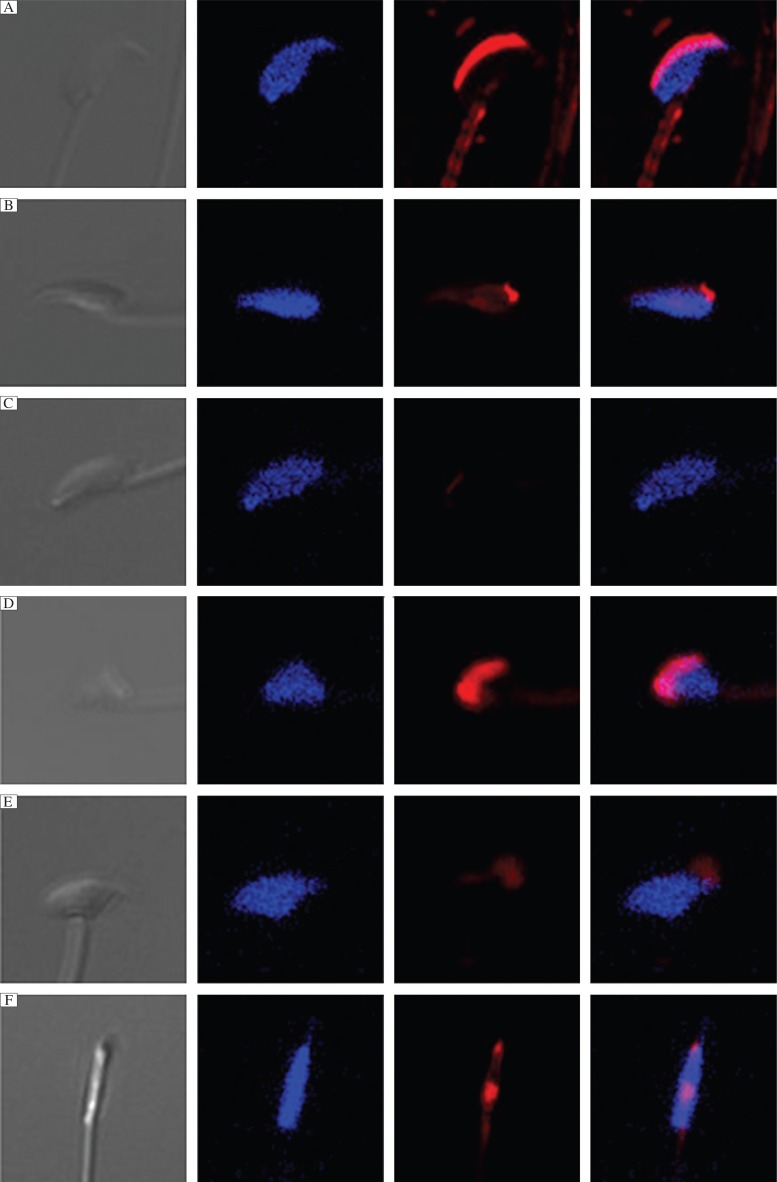
Knockdown of flotillin-2 *in vivo* induced acrosome abnormalities. Fluorescent staining of the acrosome with peanut agglutinin (red) and nuclear staining with Hoechst (blue) of normal sperm (A) and sperm with abnormal heads (B-F) from the cauda epididymis of mice injected with flotillin-2 siRNA. The sperm with abnormal heads contained an abnormal acrosome. Similar results were observed in triplicate experiments.

Equal amounts of flotillin-2 siRNA and control siRNA were separately injected into the paired testes of mice. Three w later, the sperm were collected from the caudal epididymis for analysis. Approximately 18% of sperms were abnormal in the control siRNA group; by contrast, nearly 25% sperms were abnormal in flotillin-2 siRNA-treated testes (*P* < 0.05 *vs* control, ***Fig. 6A***). We observed that a large number of sperm from the caudal epididymis of mice treated with flotillin-2 siRNA had abnormal heads and resembling irregularly shaped balls. However, these abnormalities were rarely observed in the sperm of the control siRNA group (***Fig. 6B***). The proportion of sperm head abnormalities increased markedly to 14.6% in the flotillin-2 siRNA group, which was significantly higher than that of the control siRNA group (9%, *P* < 0.05; ***Fig. 6A***). PNA staining of abnormal sperm also demonstrated various head abnormalities. The sperm with abnormal heads in the flotillin-2 siRNA group also demonstrated acrosome abnormalities (***Fig. 7A*** to ***Fig. 7F***).

## DISCUSSION

Spermatogenesis is the process of the development of sperm from undifferentiated germ cells, via a precisely-regulated well-coordinated mechanism[16],[24]. In our previous research, we combined two-dimensional electrophoresis mass spectrometry (2DE-MS) and liquid chromatography (LC-MS) to construct a series of proteomic profiles of spermatogenesis. We also obtained a complete protein profile and proteinprotein interaction map during testicular development and spermatogenesis[16]. We selected a number of these proteins for further analysis, and in this study we investigated the localization and role of flotillin-2 during spermatogenesis.

Flotillin-2 is enriched in membrane rafts. Miranda *et al*.[25] reported the localization of low-density detergent-resistant membrane proteins in intact and acrosome-reacted mouse sperm. In their research, as been enriched in membrane rafts, flotillin-2 was selected to investigate the localization in the sperm and the behavior during capacitation and the acrosome reaction. The results showed that the staining observed in the apical portion of the head before acrosome reaction extended along the acrosomal domain after acrosome reaction. This indicated that flotillin-2 may have a role in fertilization, such as sperm-egg membrane interaction. However, the mechanism is still unknown. Here, we used commercial antibody to detect the expression of flotillin-2 in mouse testis. The results showed that the antibody was very specific and only the 48 kDa band was detected. So we designed experiments to further explore the function of flotillin-2.

Along with the ongoing experiment, the results showed a new excitement. In adult mouse testes, flotillin-2 was mainly localized in pachytene spermatocytes as a strong dot-shaped signal, and was also observed in round spermatids as a sickle-shaped distribution in the acrosome during spermiogenesis. A similar distribution of flotillin-2 was observed during the first wave of spermatogenesis. Therefore, we hypothesized that flotillin-2 is not only involved in sperm fertilization as indicated[25], but may also be a critical protein during spermatogenesis, especially spermiogenesis. Therefore, we designed experiments to further explore the function of flotillin-2 during spermiogenesis.

Immunofluorescent analysis of flotillin-2 in conjunction with PNA staining demonstrated that flotillin-2 was located ahead of the acrosome, and not on the outer membrane of the acrosome. The pattern of flotillin-2 expression in the mouse testis is similar to other Golgi-related proteins. Moreno *et al*.[3] investigated dynamic acrosome biogenesis in live rhesus monkey spermatids, and observed that Golgi proteins such as Golgin-95/GM130, Golgin-97 and Golgin-160 were distributed within a spherical shape in spermatocytes and localized over the acrosomal vesicle in round spermatids. Similar results were also observed for GM130 and Golgin-97[6]. Therefore, the similar pattern of flotillin-2 expression indicates that flotillin-2 may be a novel Golgi-related protein involved in acrosome biogenesis. Here, we can explain why the expression level of flotillin-2 was decreased in adult mouse testis because another Golgi-related protein is also detected in the membranes surrounding the acrosome until the late cap-step spermatid stage, and finally is shed in the cytoplasmic droplet[3],[5],[6].

As an acrosome-related protein, we hypothesized that flotillin-2 might be involved in acrosome biogenesis. We performed *in vivo* RNAi-mediated knockdown to explore the function of flotillin-2 during acrosome biogenesis. Transcription and translation almost cease during spermiogenesis, due to the process of histone replacement by protamine[26],[27]. When siRNAs are introduced into germ cells, the siRNA will degrade its target mRNA, inhibiting the transcription of new target mRNA. Thus, the protein level of the target gene will be reduced for a relatively long period[28]. We collected sperm during the first wave of spermatogenesis three w after injection of flotillin-2 siRNA, to analyze the effect of flotillin-2 knockdown on the sperm phenotype. The rate of sperm abnormalities, especially head abnormalities, was significantly higher in the flotillin-2 siRNA-injected group than the control siRNA-injected group. Furthermore, PNA staining also demonstrated various acrosome abnormalities in the sperm with abnormal heads in the flotillin-2 siRNA-injected group.

Flotillins, also called reggies, are evolutionarily well-conserved and ubiquitously expressed from Drosophila to man[29]. They are considered to be scaffold proteins of lipid rafts and generally used as marker proteins of lipid microdomains[30],[31]. Flotillin-2 is a ubiquitous membrane protein which displays the typical cold detergent resistance of raft proteins[32],[33]. In addition, flotillin-2 is also thought to function in a number of cellular processes, including cell signaling, endocytosis and interactions with the cytoskeleton[34]. This study suggests that flotillin-2 plays a novel function during spermatogenesis. The localization and function of flotillin-2 are similar to other Golgi-associated proteins. Golgi-associated PDZ and coiled-coil motif-containing protein (GOPC) is predominantly localized at the trans-Golgi region in round spermatids. Male mice in which GOPC has been disrupted are infertile and display acrosome fragmentation in early round spermatids, with apparent abnormal vesicles which fail to fuse in the developing acrosome[7]. Hrb protein binds to the cytosolic surface of proacrosomal transporting vesicles, and lack of Hrb prevents vesicles from fusing and forming the acrosome[11],[35]. Decreased expression of flotillin-2 during the first wave of spermatogenesis leads to acrosome abnormalities, suggesting that flotillin-2 may play an important role during acrosome biogenesis, in a similar manner to GOPC and Hrb.

Acrosome biogenesis involves the transport and fusion of Golgi-derived proacrosomal vesicles to form an acrosome sac which is tightly bound to the nuclear envelope. The final shape and size of the acrosome is determined by vesicular transport shuttling from the Golgi to the acrosome[11],[36]. Although we did not detect the precise localization and mechanism of action of flotillin-2 during acrosome biogenesis, we predict that flotillin-2, as a potential novel Golgi-related protein, may interact with other Golgi-associated proteins to play an important role during mouse acrosome biogenesis. Further research will be conducted to explore the molecular mechanism of flotillin-2 during spermiogenesis and fertilization.
